# Finasteride Use Does Not Lead to Depression or Suicide: Insights From a Large‐Scale Cohort Study and Mendelian Randomization Analysis

**DOI:** 10.1111/jocd.70579

**Published:** 2025-12-02

**Authors:** Jing Wang, Daoyi Lin, Jingwei Zhao, Siyu Wei, Hongyi Wang, Weixia Li

**Affiliations:** ^1^ China‐Japan Friendship Hospital (Institute of Clinical Medical Sciences) Chinese Academy of Medical Sciences & Peking Union Medical College Beijing China; ^2^ Department of Anesthesiology China‐Japan Friendship Hospital China‐Japan Friendship Hospital Beijing China

**Keywords:** affective disorder, depression, drug safety, finasteride, post‐finasteride syndrome, suicide, UK biobank

## Abstract

**Background:**

Finasteride is widely used in clinical practice to treat androgenetic alopecia (AGA). However, its potential role in increasing the risk of depression and suicidal tendencies among users remains a topic of significant controversy.

**Patients and Methods:**

Five datasets were used as exposures and ten as outcomes. Mendelian randomization (MR) is used to evaluate the causal relationship between finasteride use and the risk of depression or suicide. We also utilized a COX regression model on the large‐scale UK Biobank cohort to explore the potential correlation between finasteride use and the risk of depression or suicide.

**Results:**

MR analysis reported that Finasteride prescription is not related to depression or suicide after the false discovery rate (FDR) correction. COX regression analysis also showed that finasteride use is not significantly associated with depression or suicide events in the UK Biobank cohort, whether or not covariates were considered.

**Conclusion:**

The existing evidence does not support the relevance between systemic use of finasteride and increased risk of depression or suicide.

## 1 Introduction

Androgenetic alopecia (AGA) is one of the most common androgen‐related diseases in the world. Not only males but also females have an AGA prevalence of over 50% by the age of 60 [1, 2]. Approved by the FDA, finasteride is now one of the most used drugs worldwide in treating disorders related to androgen, including AGA.

Despite its therapeutic benefits in treating AGA, there has been growing concern regarding the potential association between finasteride use and an increased risk of depression [3, 4]. An investigation revealed an upward trend in suicidality‐related safety signals in the U.S. Food and Drug Administration Adverse Event Reporting System (FAERS) since 2019, which peaked in 2024 [5].

However, whether finasteride use increases the risk of depression or suicide remains controversial [6]. It has been reported a lot that the incidence of depression increases significantly in finasteride users [7]. A cohort study containing 70 645 finasteride users also declares an elevation in the risk of depression or suicide after finasteride or dutasteride [8]. However, another study based on the National Veterans Health Administration administrative data of 53 848 finasteride users suggested no association between finasteride use and depression [9]. The discrepancies among previous studies highlight the need for more rigorous methodologies to elucidate the causal relationship between finasteride use and its potential mental health risks, guiding safer prescribing practices for affected patients.

To address the problems mentioned above and find solid evidence on whether finasteride will lead to depressive symptoms, a large‐scale cohort and a method that can provide a convincing correlation without bias and interference should be applied. The UK Biobank (UKB) and Mendelian randomization (MR) happen to be the perfect solution that meets all the requirements. The UKB contains massive clinical data about diagnoses and prescriptions of more than 500 thousand people. Meanwhile, using single‐nucleotide polymorphisms (SNPs) from GWAS as instrument variables (IVs), MR analysis can evaluate the causal effect of the exposure on the outcome [10].

In this study, we evaluated the potential risk of depression or suicide intention related to the finasteride prescription and performed a series of two‐sample MR analyses based on the GWAS statistic to investigate whether finasteride use increases the risk of depression or suicide, which has long been a matter of concern to dermatologists.

## 2 Materials and Methods

### 2.1 Data Acquisition of Finasteride and Depression or Suicide

The dataset of finasteride medication (ukb‐b‐2029) from the GWAS study of the MRC‐IEU consortium (https://gwas.mrcieu.ac.uk/) conducted in the European population was used as the exposure of the two‐sample MR analyses, which contained 1586 users of finasteride and 461 347 unmedicated cases as controls.

For acquiring data for the outcomes, there were several datasets whose traits contained “depression” or “suicide”. Therefore, the datasets were selected based on the following criteria: Firstly, their traits must contain “depression” or “suicide”. Secondly, the datasets with traits that contained other symptoms, such as “bipolar” and “anxiety”, were excluded. Thirdly, indirect descriptions such as “see a doctor for depression”, “Activities are undertaken to treat depression”, or “Weight change during the worst episode of depression” were excluded. Fourthly, depression datasets with a clear inducement, such as “Depression possibly related to childbirth” or “Depression possibly related to a stressful or traumatic event”, were excluded. Fifthly, the datasets containing relative information were not used as exposure. Finally, only binary datasets were used as the outcomes in the MR analyses.

To investigate the possible causal effect of finasteride indications on the outcomes, the AGA was also used as the exposure in the following MR analyses. Only one GWAS dataset contained the trait of AGA, which was employed for selecting IVs for AGA.

The details of datasets that meet all the standards above are displayed in Table 1.

**TABLE 1:** Datasets that are qualified to be the exposures or outcomes for the MR analyses.

	Name	n_case	n_control	Population
Exposure				
AGA	Androgenic alopecia	281	267 178	European
Finasteride	Treatment/medication code: finasteride	1586	461 347	European
Outcome				
Depression1	Depression	13 559	435 855	European
Depression2	Depression	23 424	192 220	European
Depression3	Depression	105 739	16 471	European
Depression4	Depression (broad)	113 769	208 811	European
Depression5	Non‐cancer illness code self‐reported: depression	19 195	317 964	European
Depression6	Non‐cancer illness code, self‐reported: depression	26 595	436 338	European
Depression7	Major depression	170 756	329 443	European
Depression8	Mental health problems ever diagnosed by a professional: depression	25 087	92 695	European
Suicide1	Suicide or other Intentional self‐harm	52 208	166 584	European
Suicide2	Ever attempted suicide	2658	2275	European

### 2.2 Study Design

**FIGURE 1:**
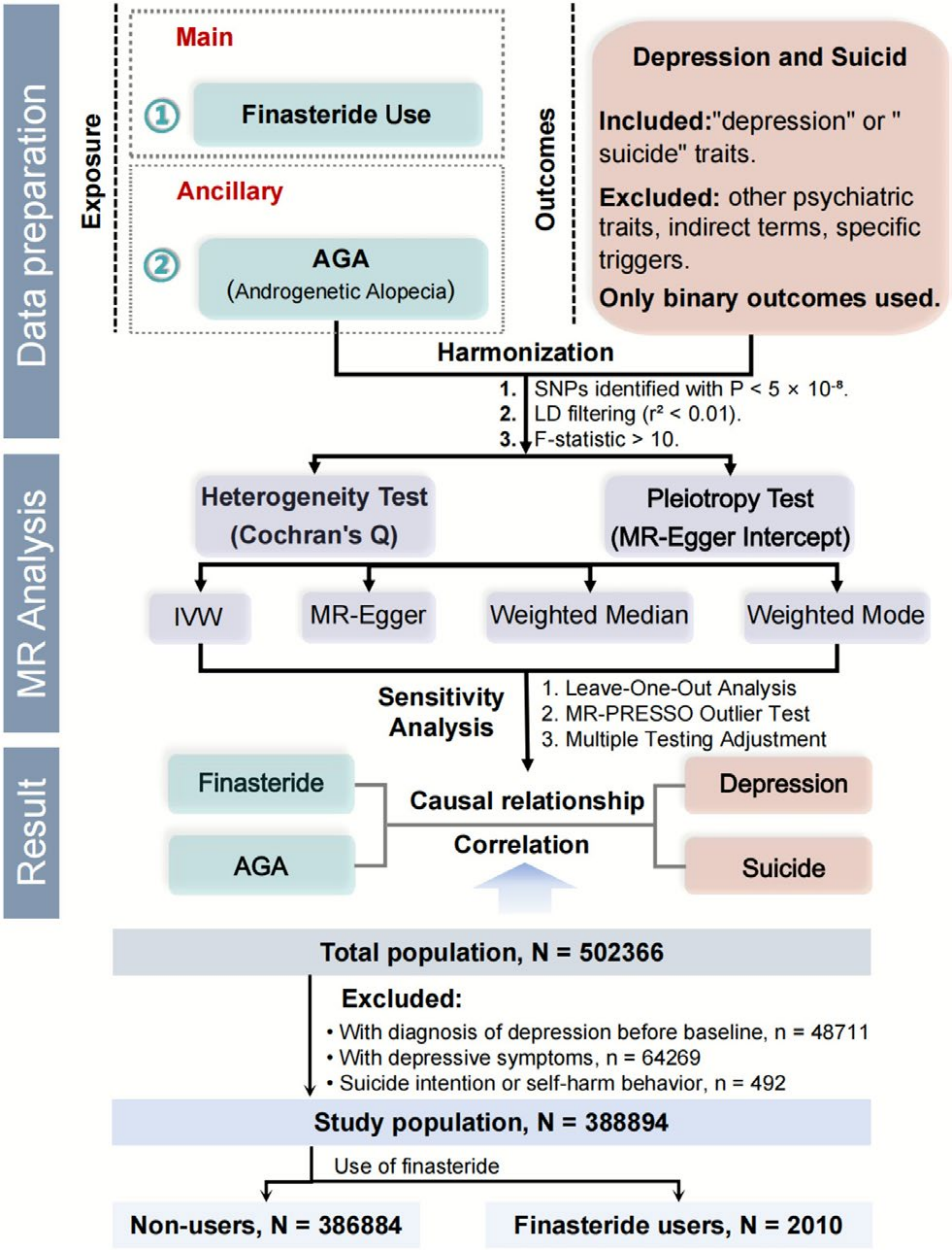
The flow chart displays the overall design of MR analysis on the previous GWAS data and UK biobank cohort study.

Based on the above data, a series of two‐sample MR analyses was conducted using the “TwoSampleMR” package. In an MR analysis, three crucial assumptions need to be met in IV selection [11]. Firstly, IVs should have a strong connection with finasteride use. Secondly, IVs should follow the random allocation rules without being affected by confounding factors. Lastly, IVs affect the outcome, which is depression in this study, only through the selected exposure and have no direct impact on the outcomes. The details of the designs and hypotheses of the MR analysis are contained in the flow chart (Figure 1).

The parameters for selecting IVs that meet all the assumptions above included a *p*‐value less than 5e‐8, an *r* ^2^ less than 0.01, and a window size of 10 Mb [12]. The inverse variance weighted (IVW) and Wald ratio methods were employed as the primary methods in MR analysis to investigate the potential causal relationship. If the exposure has more than two IVs, the MR‐Egger regression, simple mode, weighted median, and weighted mode will also be used as the auxiliary analysis methods.

Considering the existence of multiple outcomes, the potential false discovery rate (FDR) must be appropriately handled [13]. As a result, the Benjamini‐Hochberg method was employed in every analysis in our study to lower the FDR after multiple tests [14]. The significant causal relationship between the exposure and outcome was determined by a *q* value, which is the corrected *p* value after the FDR correction, under 0.05.

### 2.3 Cohort for Further Investigation

To further evaluate the potential association between finasteride use and depression, we employed a large‐scale population‐based prospective cohort in our study. Over 500 thousand adults aged between 40 and 69 were enrolled in the UKB study (UK Biobank: an open access resource for identifying the causes of a wide range of complex diseases of middle and old age) after they were fully informed and signed the consent form to participate.

Depression was defined by the ICD codes F32 and F33. Suicide was defined by ICD10 codes X60‐X84 and Y10‐34. Self‐harm (ICD codes X60‐X84 and Z91.5) was also employed as a subgroup of suicide. Finasteride users were defined by the Treatment/medication (20003) code 1140868550. The participants who had already been diagnosed with depression or had depression symptoms or suicidal intentions were excluded (Figure 1). The COX regression model was employed to analyze the data of the remaining participants.

### 2.4 Covariates

Demographic variables (age at recruitment, sex, ethnicity), social life variables (employment status, Townsend deprivation index, educational levels) and personal lifestyles (sleep pattern, smoking status, and alcohol drinking) were employed as level1 (demographic variables) and level2 (variables in social life and personal lifestyles) covariates. A detailed list of all extracted variables is shown in Table 2.

**TABLE 2:** Baseline characteristics of study population.

	Total population	Use of finasteride		*p*
		No	Yes	
	388 894	386 884	2010	
Age (years)	56.72 (8.08)	56.69 (8.08)	62.90 (5.27)	< 0.001
Gender				< 0.001
Women	204 812 (52.67%)	204 802 (52.94%)	10 (0.50%)	
Men	184 082 (47.33%)	182 082 (47.06%)	2000 (99.50%)	
Ethnicity				< 0.001
White	370 997 (95.40%)	369 041 (95.39%)	1956 (97.31%)	
No‐white	16 768 (4.31%)	16 722 (4.32%)	46 (2.29%)	
Others	1129 (0.29%)	1121 (0.29%)	8 (0.40%)	
Townsend deprivation index	−1.51 (2.97)	−1.50 (2.97)	−1.86 (2.82)	< 0.001
Educational levels				0.006
College or university degree	132 642 (34.11%)	131 889 (34.09%)	753 (37.46%)	
Below college degree	253 204 (65.11%)	251 962 (65.13%)	1242 (61.79%)	
Others	3048 (0.78%)	3033 (0.78%)	15 (0.75%)	
Employment status				< 0.001
Yes	364 155 (93.64%)	362 211 (93.62%)	1944 (96.72%)	
No	23 702 (6.09%)	23 641 (6.11%)	61 (3.03%)	
Others	1037 (0.27%)	1032 (0.27%)	5 (0.25%)	
Smoking status				< 0.001
Never smoker	215 475 (55.41%)	214 508 (55.45%)	967 (48.11%)	
Ever smoker	136 098 (35.00%)	135 182 (34.94%)	916 (45.57%)	
Current smoker	36 243 (9.32%)	36 125 (9.34%)	118 (5.87%)	
Others	1078 (0.28%)	1069 (0.28%)	9 (0.45%)	
Frequency of alcohol drink				.
Never	26 435 (6.80%)	26 298 (6.80%)	137 (6.82%)	
Less than 3 times/week	184 565 (47.46%)	183 747 (47.49%)	818 (40.70%)	
Equal or more than 3 times/week	177 756 (45.71%)	176 701 (45.67%)	1055 (52.49%)	
Others	138 (0.04%)	138 (0.04%)	0 (0.00%)	
Sleep duration				0.001
7–8 h/day	271 831 (69.90%)	270 442 (69.90%)	1389 (69.10%)	
< 7 h/day	89 350 (22.98%)	88 917 (22.98%)	433 (21.54%)	
> 8 h/day	26 549 (6.83%)	26 366 (6.81%)	183 (9.10%)	
Others	1164 (0.30%)	1159 (0.30%)	5 (0.25%)	

### 2.5 Sensitivity Analysis

The sensitivity analyses included heterogeneity and pleiotropy tests. Cochran's *Q* test was utilized to evaluate heterogeneity among instrumental variables. In the IVW approach, the heterogeneity of the IVs was statistically significant when *p* < 0.05 in Cochran's Q test. MR‐Egger and MR‐PRESSO were employed to detect pleiotropy when exposure has more than two IVs. All analyses were conducted in R Studio.

## 3 Results

### 3.1 MR Analyses Detected no Causal Relationship Between Finasteride Use and Depression or Suicide

**FIGURE 2:**
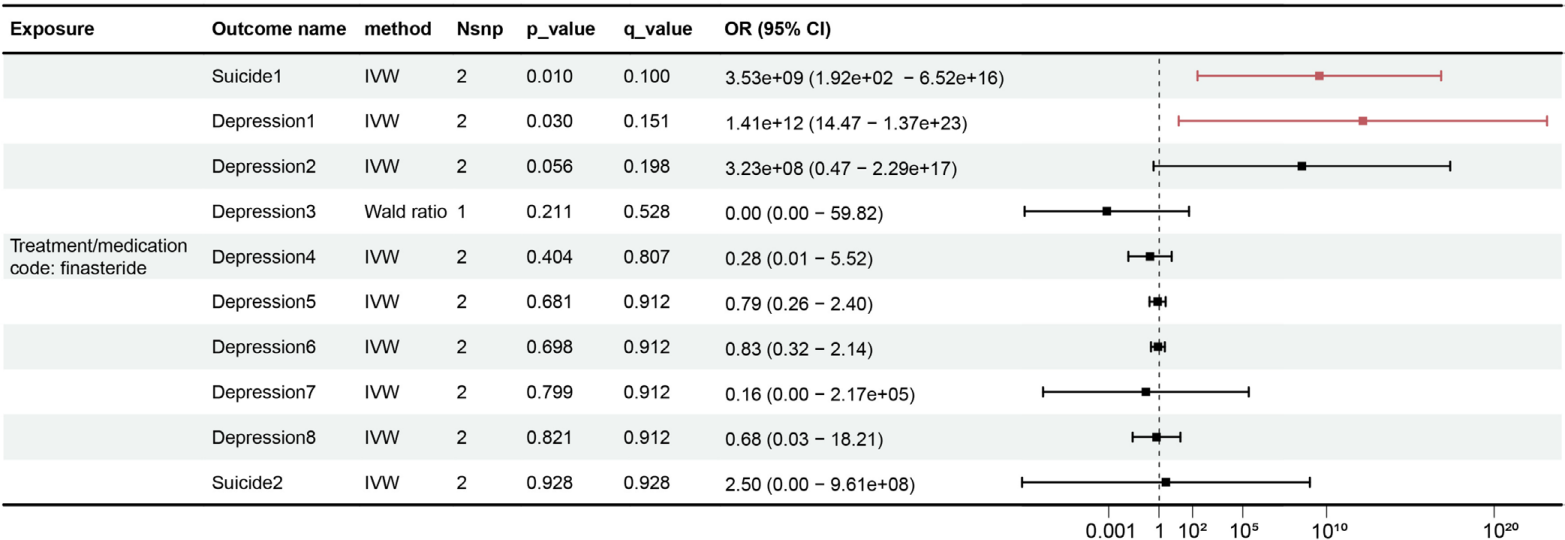
The forest plot shows the results of MR analysis after being corrected by the Benjamini‐Hochberg method (*q* value). IVW, Inverse‐variance weighted; OR, Odds ratio.

After performing a series of two‐sample MR analyses between the exposure of using finasteride and each different outcome, two outcomes were identified as the potential results of the finasteride use, including one depression outcome (Depression 1, *p* = 0.030) and one suicide outcome (Suicide 1, *p* = 0.010). The causal relationship indicated that finasteride use would significantly increase the risk of depression or suicide. However, after the FDR correction, the causal relationships were no longer significant (Figure 2).

To further investigate the potential depressive‐causing risk factors that may be associated with oral finasteride, we assessed the causal relationship between finasteride indications and depression.

To date, finasteride has been approved by the FDA for the treatment of AGA at doses of 1 mg per day. Therefore, we searched for all the publicly available statistics on GWAS related to AGA.

Only one dataset (ID: finn‐b‐L12_ALOPECANDRO) was qualified to be used as the exposure of AGA. However, under the significant level of 5e‐8, no SNP was found related to the AGA exposure (r2 < 0.01, window size = 10 000 kb).

Based on the criteria mentioned in the methods, 6 datasets were found to be qualified for the MR analyses, the information of which is displayed in Table 1.

### 3.2 Baseline Characteristics of the Enrolled Population

In the demographic variables, the finasteride users are older than the non‐users in age. The proportion of males is much higher than that of non‐users, which is in accordance with common sense, considering that finasteride is mostly prescribed to men. However, the ethnicity, as well as the variables in social life and personal lifestyles showed no significant changes despite the reported statistical difference (Table 2).

### 3.3 Association Between Finasteride Use and Depression/Suicide in the UKB Cohort

**FIGURE 3:**
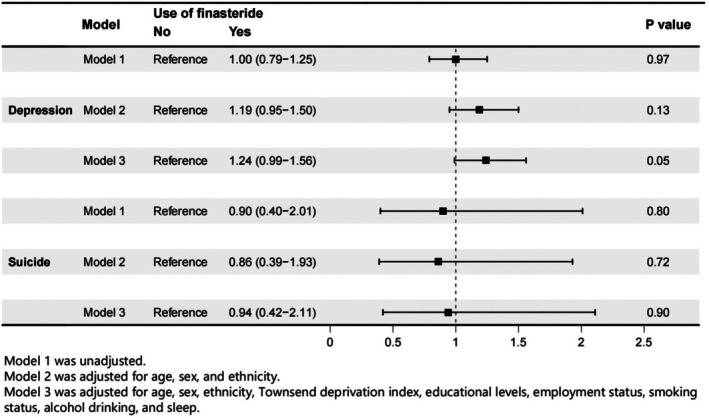
The forest plot showed the altered risk of depression or suicide between finasteride users and non‐users. The covariates that are adjusted are also shown in this plot.

The median follow‐up periods for depression and suicide were 13.7 and 13.74 years respectively. During this follow‐up period, 14 883 depression cases and 1299 suicide cases were reported in the total population. There were 2.89 newly reported depression cases in every 1000 finasteride users per year, while 2.90 were reported in the control population. Before the adjustment, the hazard ratio did not increase or decrease significantly in the finasteride users compared to the non‐users. This difference in depression risk remained insignificant after adjustment that involved two levels of covariates (Figure 3).

The incidence rate of suicide is lower than that of depression in both the finasteride group and the control group.

### 3.4 Sensitivity Analysis

No heterogeneity and pleiotropy tests were conducted for the sensitivity analysis of the causal association between finasteride use and depression or suicide due to the insufficient number of IVs.

## 4 Discussion

As one of the most used drugs in AGA treatment, the safety of oral medication of finasteride has long been a concern for dermatologists [15, 16]. In fact, the increased risk of depression has been added to the finasteride label by the FDA after 2011 because of the growing number of depression cases reported to FAERS [17].

Androgens, the leading cause of AGA, mainly exist in the form of testosterone in the circulatory system [18, 19]. It can be transferred and hydrogenated as the active form of dihydrotestosterone (DHT) by 5α‐reductase [20]. Finasteride reduces the DHT level by 60% to 70% in serum, prostate, and scalp by competitively and specifically inhibiting type 2 5α‐reductase (SRD5A2) [21].

Despite a large number of previous studies on this topic, most of the existing data come from clinical trials, the power of which could be overstated considering the limited number of cases. In addition, many factors may interfere with the incidence of diseases in a clinical trial [22]. First of all, the disease itself could also have a negative effect on a patient's mental health. One of the primary pieces of evidence that supports the connection between depression and finasteride is that the incidence of depression is higher in finasteride users than in the general population [23]. However, AGA can increase the risk of anxiety and depression in patients [24, 25]. Secondly, the negative mental effect of finasteride may be exaggerated as being spread by social media and the Internet [26]. An interesting fact is that few suicidal events were reported before depression was officially listed as one of the potential adverse effects of the finasteride label in 2011, the number of which increased exponentially after then [27]. In contrast, the adverse event ratio remains relatively low in clinical trials [17]. Therefore, it must be taken into consideration that the role of information from amateur resources such as comments on social media, could amplify the depressive emotion in finasteride users.

Our study overcame the limitation of insufficient sample size by acquiring data from the large‐scale GWAS conducted in the European population. In this study, ten datasets comprising a total of 552 990 cases of depression or suicide and 2 198 656 control cases were utilized to investigate the potential causal relationship between finasteride use and depression outcomes. Contrary to common knowledge, the causal effect of finasteride use was not determined to be significant on any depression or suicide outcome, which means taking finasteride medication for treating AGA or benign prostatic hyperplasia will not increase the risk of depression. Our finding aligns with those of some previous observational studies that refute the association between finasteride and depression [6, 28, 29]. Unlike the randomized controlled trials, the MR analysis employs genetic variants known to be closely related to the exposures as IVs to evaluate the causal relationship between the exposure and outcome, which reduces the potential of being interfered with by the confounding factors to the greatest extent. Additionally, to maintain consistency with ethnic characteristics in the exposure, only datasets from GWAS in the European population were used as outcomes, which contributes to minimizing potential bias and enhancing the credibility of the results [30].

In our study, we also analyzed the cohort of UKB to evaluate the correlation between finasteride prescription and depression or suicide, with no such evidence revealed. To the best of our knowledge, this is the first study to evaluate the potential mental risk in finasteride users based on such a large prospective cohort. By employing the covariates, we eliminated the effect of confounding factors as much as possible, making our conclusion more solid. No significant correlation between finasteride use and depression was revealed before and after the adjustment of different levels of covariates. However, it is worth noticing that after adjusting for all covariates, there was an increase in depression risk in finasteride users. Although the increase was not statistically significant, the potential risk of depression should still be noted in patients who take finasteride regularly.

Despite all the advantages, our study still has some limitations. For example, in the primary MR analysis that evaluates the potential causal association between finasteride use and depression, only one dataset of finasteride use was available as the exposure, weakening the robustness of the study. Moreover, although we employed the Benjamini‐Hochberg method for FDR correction to reduce the likelihood of false positive findings, this approach may incur the risk of overlooking a potential finasteride‐depression connection that may exist.

The results of our research warrant further validation with additional statistics from GWAS involving finasteride users. Except for the insufficient number of exposures, we did not consider the potential role of SRD5A2, the drug target of finasteride, due to the lack of available statistics. Furthermore, the currently available GWAS data originates from studies on circulating blood, where the levels of androgen‐related metabolites tend to remain stable [31]. However, a previous study found that the altered methylation level of the SRD5A2 gene in the cerebrospinal fluid may contribute more to the potentially causal effect of finasteride on depression [32]. Lastly, we were unable to compare the severity of AGA before and after taking finasteride in the UKB cohorts. Since the disease can add to the mental burden of patients, the increased risk of anxiety and depression related to the ineffectiveness of the prescription may be overlooked in our study.

## 5 Conclusion

In summary, our study offers new evidence that does not support the potentially causal relationship between finasteride use and the increased risk of depression. However, it is worth further investigating whether other factors related to finasteride use, such as targeted enzymes and androgen‐related metabolites, are causally associated with the risk of depression or suicide.

## Author Contributions

Jing Wang and Daoyi Lin were responsible for conceptualization, data curation, investigation, and writing the original draft. Jing Wang contributed to methodology and software development. Jing Wang and Jingwei Zhao carried out the investigation. Siyu Wei and Hongyi Wang validated and visualized the results. The project was supervised and funded by Weixia Li. Hongyi Wang and Weixia Li also reviewed and edited the manuscript.

## Funding

This research was funded by the National High Level Hospital Clinical Research Funding (2025‐NHLHCRF‐PY‐18) and China‐Japan Initiated Trial Project and Friendship Hospital Investigator (2024‐HX‐79).

## Ethics Statement

The authors have nothing to report.

## Consent

The authors have nothing to report.

## Conflicts of Interest

The authors declare no conflicts of interest.

## Data Availability

The data that support the findings of this study are available from the corresponding author upon reasonable request.
